# Identification and Applications of Key Elements in the Development of Pictorial Support for People With Aphasia After Stroke: A Co‐Design Approach

**DOI:** 10.1111/hex.70478

**Published:** 2025-10-29

**Authors:** Malin Bauer, Monica Blom Johansson, Ellika Schalling, Emma Kjörk

**Affiliations:** ^1^ Department of Public Health and Caring Sciences, Speech‐Language Pathology Uppsala University Uppsala Sweden; ^2^ Department of Rehabilitation Medicine Danderyd University Hospital Stockholm Sweden; ^3^ Geriatrics, Rehabilitation Medicine and Pain Center Uppsala University Hospital Uppsala Sweden; ^4^ Department of Clinical Neuroscience, Institute of Neuroscience and Physiology, Sahlgrenska Academy University of Gothenburg Gothenburg Sweden; ^5^ Centre for Person‐Centred Care (GPCC) University of Gothenburg Gothenburg Sweden

**Keywords:** aphasia, co‐design, person‐centred care, pictorial support, stroke

## Abstract

**Background:**

Access to health information is essential to ensure safe, person‐centred care and shared decision‐making. Following a stroke, communication difficulties, such as aphasia, often cause barriers to communication with healthcare staff and risk worsening the quality of care. Adaptations to make written information more accessible for persons with aphasia often include pictures. However, guidelines on creating pictorial support for persons with aphasia are limited. This study aimed to (1) identify key elements to consider when developing pictorial support to aid communicative accessibility and (2) co‐design an accessible pictorial support for the follow‐up tools, Post‐Stroke Checklist and the pre‐visit tool Stroke Health, together with stakeholders.

**Methods:**

Six persons with aphasia, a patient‐partner with stroke, and eighteen stroke healthcare professionals were involved to co‐design a pictorial support. Stakeholders were engaged via interviews, e‐mail surveys and consensus workshops. Data were analysed using reflexive thematic analysis.

**Results:**

A reflexive thematic analysis resulted in two main themes and four sub‐themes. First, ‘Aspects to consider in the design of pictures in pictorial support’: *The importance of being able to relate to pictures* and *Interpretation of pictorial support is complex and pictures risk becoming barriers if not carefully illustrated*. Second, ‘The contextual use of pictorial support’: *Pictures must correspond closely to the text*, and *Communication partner skills are important to enhance the use of pictorial support*. The analysis also resulted in a list of key elements to consider when developing pictorial support.

**Conclusions:**

Findings highlight the importance of pictures being relatable and closely matching the written information. Identified key elements can be used as principles in the future development of pictorial support in different settings.

**Patient or Public Contribution:**

Both persons with lived experience of stroke and aphasia and healthcare professionals within stroke rehabilitation contributed to the design process. This involvement included individual interviews, e‐mail surveys and one consulting group discussion. A patient‐partner with stroke was engaged in all workshops where feedback was discussed before deciding on revisions to proceed with. The final version of the pictorial support has been presented to stakeholders at a patient organisation, contributing to the recruitment of participants and facilitating rooms for interviews.

## 1 Introduction

People who have suffered a stroke report a great need for understandable information about stroke, including medication and treatment options [1]. Following a stroke, communication difficulties are common, and aphasia affects approximately one‐third of patients [2]. Aphasia frequently causes barriers to the delivery of healthcare services and hinders access to healthcare‐related information and communication [3, 4, 5]. Consequently, patients with aphasia after stroke face an increased risk of distress, adverse events and insufficient planning for discharge [6].

The provision of accessible health information has been identified as an important factor in the development of equitable healthcare systems [7]. Health literacy, defined by Sørensen et al. [8] as ‘people's knowledge, motivation and competence to access, understand, appraise and apply health information’, is fundamental to an individual's ability to access and engage in healthcare. Empirical studies have shown that individuals with low health literacy have difficulties understanding health information and experience poorer health outcomes [7]. Although health literacy embodies literacy and cognitive abilities, it should be understood as a socially situated practice [9]. This perspective constitutes a shift from a focus on personal skills to health literacy as a dynamic phenomenon, that varies between individuals, situations, cultures and contexts [9, 10]. Consequently, healthcare providers have a great responsibility to deliver information in an accessible and customised manner.

The inclusion of pictures in written material directed to people with aphasia is a common strategy to increase accessibility [11]. However, research supports the inclusion of pictures in health information in a broader sense, as it may be beneficial for patients in general, and especially important for increasing understanding among individuals with low health literacy [12]. In the development of pictures, providers of health information are advised to consider their target group and, whenever possible, include them in the development process [13]. However, with only a few exceptions [14, 15], the majority of materials directed to people with aphasia have been developed without engaging stakeholders.

Improving the accessibility of written information for people with aphasia involves attending to design principles regarding font size, line spacing, document length and the inclusion of graphics. There is evidence that individuals with aphasia appreciate these kinds of adaptations [15]. A possible reason may be that adding images to written information may contribute to a more accessible appearance, which might encourage attempts to read it [11]. Existing research on the impact of images on reading comprehension is inconclusive [11, 16, 17]. However, research in this area is limited and there is a lack of guidelines on how to construct images that are easy to interpret and support understanding for persons with aphasia. One initiative to construct such criteria encourages clinicians to consider the amount of content, context and positioning of human figures in the selection of pictures [18]. Principles such as consistently depicting a concept across the material and avoiding ambiguities have also been reported as important [14].

Disabilities following a stroke are often complex and long‐term. Many patients experience stroke‐related problems or unfulfilled needs related to body function, activity and participation, or environment years after falling ill [19]. Improved continuity of care and efforts to increase stroke‐specific health literacy have been suggested as possible solutions to counteract patients' feelings of abandonment and lack of knowledge and skills to re‐engage [20]. The Post‐Stroke Checklist (PSC) [21] is a standardised tool for structured follow‐up, developed to assist healthcare professionals in identifying stroke‐related problems and facilitate referral for treatment. The PSC is endorsed by the World Stroke Organisation and covers topics related to mobility, cognition, communication, secondary prevention, relationship with family, and mood [21]. To offer patients the possibility to prepare before a healthcare visit, the pre‐visit tool Stroke Health was recently developed through a co‐design process with stakeholders [22]. Stroke Health is a validated questionnaire based on the questions in PSC to facilitate shared decision‐making. The questions from PSC are presented along with patient‐directed texts with brief descriptions and examples of each topic. Although the PSC is a tool for dialogue, previous research suggests that healthcare professionals' communication skills are still key to the successful use of the tool and the identification of problems and needs related to health [23]. However, to date, there is no adaptation of PSC or the related pre‐visit tool Stroke Health to support communication between healthcare professionals and persons with cognitive and/or communication difficulties after stroke.

To sum up, communication difficulties after stroke risk worsening the quality of care and may even cause maltreatment. Pictorial support is often suggested and preferred by patients as a strategy to support communication. However, guidelines on how to construct images are limited. This study aimed to (1) identify key elements to consider when developing a pictorial support to aid communicative accessibility and (2) co‐design an accessible pictorial support for the follow‐up tools PSC and the pre‐visit tool Stroke Health, together with stakeholders.

## 2 Methods

### 2.1 Overview

The design process was influenced by principles for service design [24]. Service design is a holistic and human‐centred approach, hence well‐suited to the development of healthcare services. Inherent to service design is a co‐creative approach often involving the use of prototypes in an iterative process of testing, reflecting, interpreting feedback and devising new prototypes for services or products [25]. The current study also aligns with the framework for the development of complex interventions [26], focusing on the phase of development, specifically the development of an aphasia‐friendly version of the pre‐existing PSC and the related pre‐visit tool Stroke Health. To deepen our understanding of what persons with lived experience of aphasia and speech‐language pathologists (SLPs) value as key elements in accessible pictorial support, their responses from individual interviews and e‐mail surveys were analysed in a reflexive thematic analysis [27].

### 2.2 Co‐Design and Stakeholder Engagement

The varied use of the term *co‐design* across healthcare research risks conceptual ambiguity [28]. To ensure clarity, in this paper, we use the term co‐design to describe stakeholder engagement in the iterative design process of developing a pictorial support. We acknowledge that the level of stakeholder engagement can be characterised along a continuum and may vary in different parts of the project [29, 30]. In this study, various stakeholders were consulted repeatedly, guiding the design process. Stakeholders included both persons with lived experience of stroke and aphasia, SLPs and other healthcare professionals with expertise in stroke rehabilitation.

**Figure 1:**
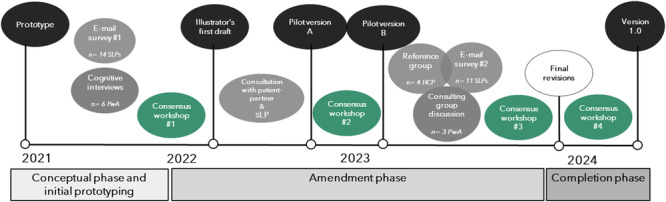
Illustration of the iterative design process. PwA = person with aphasia, SLP = speech language pathologist, HCP = healthcare professional.

Throughout the process, four consensus workshops were conducted via the digital platform Zoom (Figure 1, green circles). The workshops aimed to determine amendments and revisions based on stakeholder feedback. The first author (M.B.), the last author (E.K.) and a patient‐partner with stroke participated in all sessions. The patient‐partner contributed insights on the pictorial content, informed by the lived experience of stroke, and was actively involved in decisions, thereby exemplifying participation throughout the design process [29]. Before each workshop, the first author (M.B.) collected and condensed recent feedback from stakeholders and presented this comprehensibly using Microsoft Excel. This opportunity for individual preparation was important to ameliorate power balance. Workshops facilitated the sharing and discussion of ideas, with human‐centredness [24] maintained by consistently referencing stakeholder input to guide all design decisions. Any differing viewpoints were addressed by prioritising the overall context of the pictorial support and reviewing evidence for aphasia friendliness [14, 15]. The thematic analysis helped highlight the essence of participant feedback obtained via a combination of surveys and interviews and to achieve consensus. Each consensus workshop was summarised in a structured document specifying requests and modifications for the project illustrator, who subsequently incorporated the requirements into new versions of pictorial support.

### 2.3 Participants and Recruitment

#### 2.3.1 Persons With Aphasia

Six persons with aphasia (Table 1) were included in individual face‐to‐face interviews. They were recruited from an aphasia centre and purposively sampled to represent men and women of different ages with experience of living with aphasia. Their aphasia symptoms included word‐finding difficulties and a need for the interviewer to use supportive strategies in conversation. All participants had functional reading ability. Inclusion criteria were: chronic aphasia > 6 months post‐stroke and adequate hearing and vision to enable participation. Exclusion criteria were: severe aphasia and/or cognitive impairment hindering the ability to give informed consent, even after information was provided using communicative support.

**Table 1:** Background information about participants with aphasia.

ID	Contribution during the design process	Age	Gender	Time since onset (in years)
PwA 1	Individual interview	85	Male	3
PwA 2	Individual interview and consulting group discussion	41	Female	14
PwA 3	Individual interview	56	Male	5
PwA 4	Individual interview and consulting group discussion	48	Male	3
PwA 5	Individual interview	62	Male	4
PwA 6	Individual interview and consulting group discussion	48	Female	16

Abbreviation: PwA = person with aphasia.

Three participants with aphasia were also invited to participate in a consulting group discussion during the final stage of development, to explore acceptability and final modifications. The number of participants in the consulting group discussion was limited to ensure that everyone had an opportunity to speak. These participants were purposely sampled based on their ability to provide rich feedback in the initial interviews.

#### 2.3.2 SLPs

Fourteen SLPs (12 female and 2 male) were included via e‐mail surveys with open questions about the content of the pictorial support. They were recruited and contacted via e‐mail or face‐to‐face through contacts in stroke rehabilitation. All SLPs had between 5 and 30 (median: 12.5) years of experience in stroke care and rehabilitation. This group was consulted twice during the design process.

#### 2.3.3 Patient‐Partner With Stroke

The patient‐partner had aphasia in the initial period following the stroke, but later recovered. He still experienced mild word‐finding difficulties and fatigue. The patient‐partner was engaged in a patient organisation and had previously contributed to the development of the pre‐visit tool Stroke Health.

#### 2.3.4 Reference Group of Healthcare Professionals

Two physicians, one occupational therapist and one physiotherapist provided feedback during the design process. These professionals were purposely recruited since they had extensive experience in stroke rehabilitation (25–45 years, median: 33.5) and had previously contributed to the development of both PSC and Stroke Health. The feedback from this reference group was collected face‐to‐face, noted and discussed during consensus workshops.

### 2.4 Procedure and Development

#### 2.4.1 Conceptual Phase and Initial Prototyping

The project started by acknowledging the need for additional support for communication, to complement the PSC, as suggested by Kjörk et al. [23]. To create a prototype of pictorial support, images were retrieved from an open‐access image bank. Consistency in style and design was the aim. However, more than one image system was used to select images to best illustrate the topics in the PSC and the pre‐visit tool Stroke Health.

Initially, persons with aphasia were interviewed to capture the participants' spontaneous responses and immediate interpretations of the pictures in the prototype. Additionally, feedback from SLPs was collected through an e‐mail survey.

#### 2.4.2 Amendment Phase

The amendment phase began with the first consensus workshop (Figure 1), during which feedback on the prototype from SLPs and persons with aphasia was summarised and discussed. A list of requirements and requests for revisions, based on their responses, was then sent to the illustrator. The illustrator initially completed a limited selection of pictures to suggest and exemplify the manner and style of the new pictorial support. The patient‐partner and an SLP with extensive experience in aphasia rehabilitation were consulted on this first draft of pictures. Their feedback was relayed back to the illustrator before pilot version A of the full material was completed. Pilot version A was reviewed and discussed in the second consensus workshop before new requests for revisions were sent to the illustrator.

Approaching the final result with Pilot version B, another round of wide stakeholder input was initiated. E‐mail surveys were again sent to SLPs, a consulting group discussion with persons with aphasia took place, and a reference group of other healthcare professionals with experience in stroke was consulted face‐to‐face. This new input was gathered to validate the latest version of the pictorial support and to inform the upcoming consensus workshop before being relayed to the illustrator for final review and revisions.

#### 2.4.3 Completion Phase

In the last phase of the design process (Figure 1), version 1.0 of the pictorial support was completed after some last revisions and amendments. Closing reflections and future considerations were discussed in a final consensus workshop.

### 2.5 Data Collection

#### 2.5.1 Cognitive Interviews

In the initial conceptual phase, individual interviews with six persons with aphasia were conducted using a cognitive interview technique commonly characterised by interviewing a small, purposive sample to reveal the participants' cognitive process [31]. Cognitive interviews are frequently employed in survey development to gain insight into how informants interpret and reason about questions [32].

The focus of the interviews was on the participants' reactions to and opinions about the prototypical images in the pictorial support. The images from the prototypic pictorial support and patient‐directed texts from the pre‐visit tool Stroke Health were presented on a computer in front of the participant. Participants were encouraged to ‘think aloud’, and the interviewer supported them by probing for more elaborate responses. Established and evidence‐based supportive communication strategies for persons with aphasia were used throughout [33]. All interviews took place at an aphasia centre. At the end of each interview, the first author (M.B.) summarised the content of what had been said. This was done to ensure shared understanding and credibility.

#### 2.5.2 E‐Mail Surveys

E‐mail surveys were sent to SLPs twice in the design process. In the initial conceptual phase, the responses to the pictures in the prototype were collected. During the amendment phase of the study, the e‐mail survey collected insights and propositions for revisions on pilot version B.

In the first e‐mail survey, 14 SLPs were included to provide feedback on the prototype. Participants had access to both the PSC and the pre‐visit tool Stroke Health when assessing and commenting on the pictures in the prototype. A demo version of Stroke Health, accompanied by pictorial support, was accessed via a link to the digital platform Healthcare Guide 1177.se, and the PSC was sent as an attached file. In the e‐mail survey participants were asked to (1) identify possible problems or barriers that could hinder interpretation of the images and (2) provide feedback on how to improve/adjust images.

In the second e‐mail survey, 11 of the SLPs from the first survey provided feedback. All participants had access to pilot version B when providing their responses and insights. Participants were asked to reflect on the same questions as in the first survey.

#### 2.5.3 Field Notes

Stakeholder input collected face‐to‐face during the amendment phase through consultation with an SLP and the patient‐partner, the reference group, and the consulting group discussion was documented using field notes. Data collected from the second e‐mail survey and field notes were not included in the thematic analysis. Still, they were used to explore acceptability and validate the new version of the pictorial support.

### 2.6 Analysis

#### 2.6.1 Reflexive Thematic Analysis

Data were collected through cognitive interviews and e‐mail surveys. Data were initially transcribed, coded and analysed separately before merging the two datasets in one joint analysis, including the perspectives of both persons with aphasia and SLPs. Coding and analysis were guided by Braun and Clarke's guidelines for reflexive thematic analysis [27, 34]. Familiarisation was achieved through transcribing interviews verbatim and compiling and rereading the responses to the e‐mail survey (M.B.). Due to inherent communication difficulties, the interviews were video‐recorded to include nonverbal communication and the use of communication strategies in the analysis. Transcriptions of nonverbal communication were included, for example, gestures replacing spoken words, pointing to pictures or facial expressions. An inductive approach was taken where codes were generated directly from the data. Due to word‐finding problems and misunderstandings leading to the need for repairs in conversation, the coding of the interviews sometimes consisted of larger chunks of data. Coding moved between a semantic and latent level, including both what participants said explicitly and what was understood by underlying meanings. Semantic content was valuable as concrete feedback on specific pictures, whereas the latent codes formed overarching themes that later guided the iterative design process in developing new pictures. Codes were generated and grouped into initial conceptual themes (M.B.) and discussed and reviewed in workshops with the research team (M.B., M.B.J., E.S. and E.K.) to ensure internal homogeneity and external heterogeneity across the dataset. Refining and defining the final themes were made through consensus in the research team (M.B., M.B.J., E.S. and E.K.). Both NVivo and MS Office software were used in data management and analysis.

#### 2.6.2 Reflexivity

All participating researchers have clinical experience working with stroke rehabilitation. As clinicians and researchers, we have experienced both the utility and limitations of pictorial support. The first author (M.B.) conducted all interviews. She is an SLP with long experience in aphasia rehabilitation and strategies to support conversation in aphasia [33]. This background and understanding directed attention to the perspectives of persons with aphasia and SLPs as stakeholder partners in co‐design research. The inherent language disorder affected the entire process from planning and conducting the interviews with persons with aphasia to coding and analysis. The importance of taking a more active role and using supportive communication techniques when interviewing persons with aphasia has previously been described as essential to facilitate conversation and to reveal participants' competence [35, 36, 37].

### 2.7 Ethical Approval

Ethical approval was obtained by the Swedish Ethical Review 2017‐08‐29; 556‐17 and 2022‐01‐11; 2021‐06723‐02.

## 3 Results

The analysis resulted in two main themes and four sub‐themes described in detail below (Tables 2 and 3). The results highlight aspects of the design and use of pictorial support, presented from the perspective of persons with aphasia and SLPs in a joint analysis. Participants contributed ideas and concrete suggestions, which were directly incorporated into the iterative design process and the development of new pictures. Hence, the analysis also resulted in a list of key elements (Tables 2 and 3) that were closely considered when developing the new pictorial support.

**Table 2:** First main theme, quotes, sub‐themes and key elements linked to these themes.

Quotes	Sub‐themes	Main theme	Key elements to consider
‘Some people are partly paralyzed, I don't know, maybe you could have a shopping trolley like this (showing a trolley being pushed using only the left hand)’. [PwA id 7] ‘You can have pictures, but not so “childish” (air quotes), this type of pictures, but not this “childish” (air quotes). This looks like a three‐year‐old’. [PwA id 7] ‘I would like the material to represent diversity in culture and ethnicity. Perhaps one of the characters could have dark skin or someone could wear a hijab?’ [SLP id 4] ‘What about old people? There should be something for pensioners too’. [PwA id 1]	Importance of being able to relate to pictures	Aspects to consider in the design of pictures in pictorial support	✓ Design pictures with the target audience in mind— adult manner to adults ✓ Display heterogeneity of condition ✓ Make pictures relatable by a diverse representation of age, gender and ethnicity ✓ Emphasise detailed facial expressions ✓ More context and details may ease interpretation
‘The use of pictures always risks leading patients to think of something specific, and thereby losing out on information that they might have shared. In all cases, some characteristics must be chosen to illustrate these types of concepts. Hopefully, patients can see them as just examples of a broader concept’. [SLP id 11] ‘More like a picture illustrating a facial expression (mimicking pain and touching her arm). Ow, ow, more like that. Ouch, (repeating the pose) like that’. [PwA id 7] ‘The picture is possibly a bit unclear since the person expresses confusion rather than pain’. [SLP id 8]	Interpretation of pictorial support is complex and pictures risk becoming barriers if not carefully illustrated		

**Table 3:** Second main theme, quotes, sub‐themes and key elements linked to these themes.

Quotes	Sub‐themes	Main theme	Key elements to consider
‘Let's see, “do you experience any other problems after your stroke that affects your recovery or gives you problems” bam bam. Here comes question after question’. [PwA id 6] ‘Hm, do you wish to bring up something else? What could that be? I have difficulties with this stuff. I find it much easier to talk about stuff that is already there. If I only have open questions, I just: “I don't know.” Surely there are things, but I can never think of them’. [PwA id 5] ‘I want both text and pictures. It's much easier. And I have fatigue so I have to think about each question and read again’. [PwA id 7] ‘I wouldn't be helped by any of the pictures alone. You need the text. Something to say what it's about’. [PwA id 5]	Pictures must correspond closely to the text	The contextual use of pictorial support	✓ Pictures and text must closely align
‘The pictures are good. However, the clarity is very dependent on the person presenting the pictures’. [SLP id 11] ‘It would be great if the personnel were offered some form of education or introduction before using the material’. [SLP id 11] ‘I think it would help many nurses to realize the importance of using the pictures themselves, to invite patients to point, ask for clarifications, etc’. [SLP id 13]	Communication partner skills are important to enhance the use of pictorial support		

### 3.1 Aspects to Consider in the Design of Pictures in Pictorial Support

In the first theme, participants described features and aspects related to the design of pictures. This included the relatability and manner of the pictures, as well as the difficulties associated with the design of pictorial support.

#### 3.1.1 Importance of Being Able to Relate to Pictures

Persons with aphasia emphasised the importance of pictures in pictorial support being relatable to the target audience. In this particular material, the importance of making stroke visible, avoiding a childish appearance, and a diverse representation was especially stressed. One person with aphasia described that she did not relate to pictures displaying two‐hand activities since having hemiparesis after her stroke. Pictures did not seem relatable when drawings were too childish in their appearance. The results suggest that drawings may be used in pictorial support for adults; however, an awareness of a style and design when directed to adults is needed. Persons with aphasia also wanted the images to reflect the heterogeneity in stroke symptoms and severity, as described here, when discussing pictures of mobility aids and wheelchairs to illustrate mobility: ‘It could be something simpler, that you can walk with a cane, a walking stick, or something simple, not too complicated’ [PwA id 1].

The importance of diverse representation in culture, gender, sexuality and age across the material as a whole was raised by both persons with aphasia and SLPs.

#### 3.1.2 Interpretation of Pictorial Support Is Complex and Pictures Risk Becoming Barriers If Not Carefully Illustrated

Pictures could sometimes mislead interpretation by being unclear or ambiguous. The difficulty of illustrating abstract and broad concepts was raised by both persons with aphasia and SLPs. Individual preferences and life situations are highly varied and can be challenging to capture in a single picture to illustrate questions about life after stroke. SLPs emphasised that, while several pictures may be required to fully illustrate a concept, too many pictures and details may cause a busy and cluttered appearance. Furthermore, while concrete examples may help demonstrate an abstract concept, there are also risks of narrowing the scope of the question and leading to a more literal interpretation. This became apparent in the interviews with persons with aphasia when confronted with pictures of a doctor, a speech bubble and a packet of cigarettes, illustrating the topic of health advice in the prototype. This illustration misled many participants into thinking the question was solely about smoking habits.

The addition of more details and context was suggested by both persons with aphasia and SLPs as a way to support the interpretation of pictures. This included illustrating whole bodies rather than body parts, as well as conveying concepts such as stress and pain through detailed facial expressions. ‘Make it clear that the question is about difficulties (post‐stroke). Adjust the facial expressions to convey that the person finds the activities hard. And illustrate in some way that the person is struggling’ [SLP id 4]. SLPs believed that poorly or ambiguously depicted facial expressions could hinder understanding.

### 3.2 The Contextual Use of Pictorial Support

In the second theme, participants described aspects related to the context of pictures and the use of pictorial support. Here, it became apparent that pictorial support is part of a palette where written information and the clinician's communication skills all play a crucial role in providing accessible information.

#### 3.2.1 Pictures Must Correspond Closely to the Text

All participants emphasised the importance of pictures and text to closely align. If a text states several examples, it is important that all of the examples have a corresponding picture and preferably occur in the same order they appear in the text.

The use of plain language was favoured across participants. Many of the SLPs noted that, in the case of the PSC, the questions are formulated in a way that may lead to misunderstandings and false answers throughout the questionnaire. ‘I have mostly reacted to how the formulations, in combination with the pictures, might confuse patients and thereby the validity of answers. Questions stated as: ‘Are you finding it more difficult to…’ pose the risk of being interpreted as ‘Are you able to…’' [SLP id 4]. To increase accessibility for persons with aphasia, SLPs expressed a need to review the layout and design in terms of spacing, font size and making response alternatives and checkboxes visually apparent. This includes wording and formulation of questions. The use of merged questions and open‐ended questions to invite the respondent to add to the questionnaire was raised as a barrier by persons with aphasia. Instead, multiple choice or lists of examples were suggestions of support. Lastly, respondents raised the need for an additional response alternative, such as ‘don't know’.

Although all persons with aphasia in this study had functional reading ability, all preferred the addition of pictures. Participants talked about how pictures enhanced, confirmed and clarified the messages in the text. However, it was expressed that pictures must be presented in context and in combination with the text. Beyond supporting reading comprehension, pictures appeared to help navigate the text and make reading easier. One participant explained how pictures can be invaluable in fatigue.

#### 3.2.2 Communication Partner Skills Are Important to Enhance the Use of Pictorial Support

Ultimately, participants acknowledged that pictures alone may not be sufficient to support understanding. Rather, it is the clinician's communication skills and the practical use of pictures in interaction that will support patient–provider communication. Clinicians may need to use additional communication strategies where there is a lack of clarity or ambiguity.

SLPs contributed with several areas for future development and clinical implications. Many noted that clinicians may need an introduction to using pictorial support in conversation. Additionally, supplemental material with pictures to support conversations around follow‐up questions was suggested. When using the pre‐visit tool Stroke Health in its digital form, features such as text‐to‐speech were proposed to increase accessibility.

### 3.3 The Design Process and the Pictorial Support

**Figure 2:**
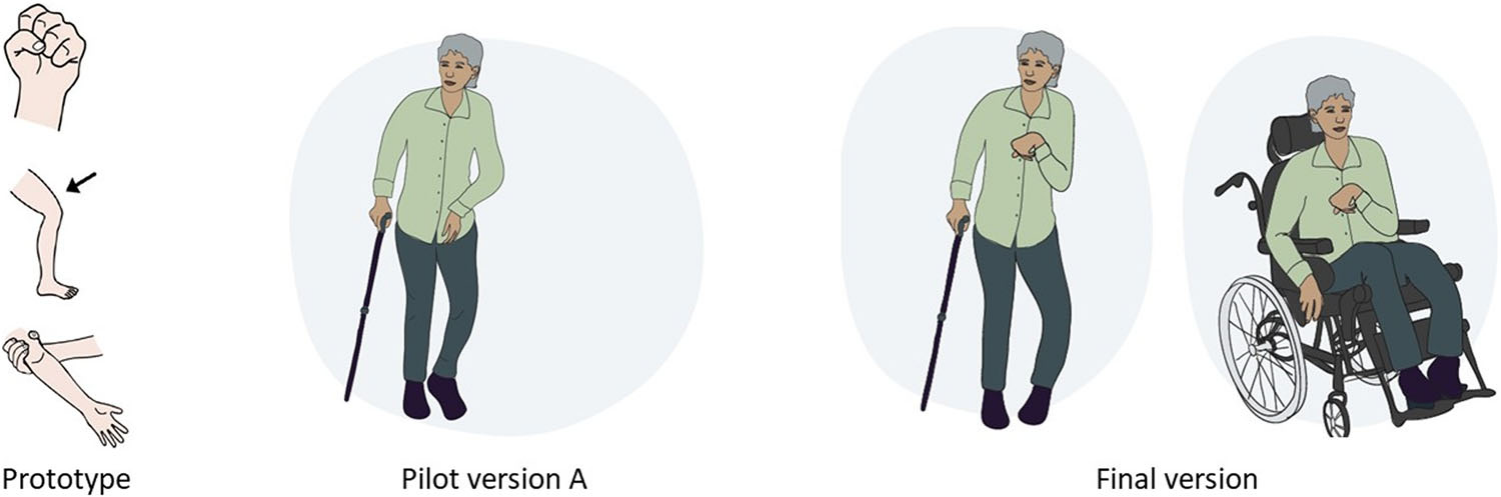
Example of the development of the image illustrating spasticity, from prototype, pilot version A and final version.

**Figure 3:**
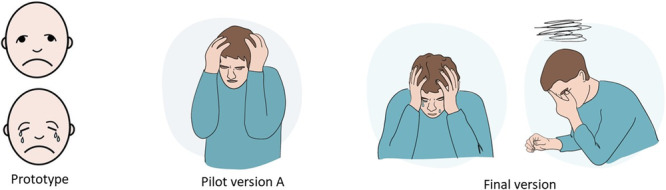
Example of the development of the image illustrating mental health, from the prototype, pilot version A and final version.

The new pictorial support aimed to incorporate the insights from the reflexive thematic analysis and the key elements in the pictorial support. The new pictures were designed in an adult manner and displayed a broad spectrum of stroke symptoms and severity. To illustrate the explicit use of stakeholder feedback, the pictures of spasticity and mental health may serve as an example (Figures 2 and 3). In the prototype, spasticity was illustrated by a closed hand, a leg and an arm in a stretched position. This caused misleading associations, as described in this quote: ‘This hand, it looks like someone is about to fight (laughs)’ [PwA id 2].

In pilot version A, more context was added to the illustrations throughout the pictorial support, as opposed to only presenting an isolated hand, arm or face. To account for the wide range of stroke severity, and to further increase relatability, the picture illustrating spasticity in the final version incorporates both a person walking and a person in a wheelchair (Figure 2).

The importance of carefully illustrated facial expressions, as highlighted by both SLPs and persons with aphasia, was also incorporated in the new version (Figure 3). The new pictures aimed for a diverse representation of age, gender and ethnicity.

The final version of the pictorial support is now available and can be used freely in clinical settings. The pictorial support integrated in PSC and Stroke Health is available in paper format, see Appendix 1 and 2. Stroke Health is also available in digital form.

## 4 Discussion

This study was motivated by the overarching objective to overcome challenges in communication between healthcare professionals and persons with aphasia after stroke. With an inherent language disorder, persons with aphasia are particularly vulnerable when it comes to patient–clinician communication. However, this study shows that, despite all having functional reading ability, participants valued the addition of pictorial support. The results suggest that pictures may serve as cognitive support and be helpful to counteract fatigue. This finding aligns with previous research, suggesting that the use of pictures has the potential to support a wide range of patients, particularly those with low health literacy, to better understand health information [12].

A key finding was the importance of being able to relate to the images, both in terms of personal attributes and stroke symptoms. The possibility of recognition in terms of diverse representation in age and ethnicity in images has been previously reported [13]. Furthermore, our results underline the risk of misdirected pictures being seen as childish or degrading. This issue and the importance of exploring preferences when selecting pictures for an adult population have previously been raised [15], reinforcing the significance of creating pictures to fit the target audience.

In our study, participants expressed that pictures depend on the text to provide information and context. For persons with aphasia who experience reading difficulties, assistance may be required, either from another person or through digital means. Congruence between text and images was essential for both persons with aphasia and SLPs. This finding is also in line with previous research [14]. Illustrating wide‐ranging and varied concepts and topics proved to be challenging. The results highlight both the risk of images limiting the scope of the question if they are too specific, but at the same time, the risk of being difficult to interpret and relate to if they are too general. This act of balance requires careful consideration and close collaboration with stakeholders throughout the design process.

The results also highlight the importance of communication to be flexible and person‐centred. While the use of open‐ended questions may be a way to initiate dialogue with some patients, it may become a barrier for persons with aphasia. In this study, both persons with aphasia and SLPs agree on the utility of pictorial support, although it may not be enough to support conversations fully. Although the inclusion of pictorial support was solely received as positive, the SLPs strongly emphasised the need for additional communication strategies and possibly an introduction to healthcare staff on how to use pictorial support in conversation. This finding is significant since healthcare providers' communication skills are essential in the implementation of person‐centred care [38].

At the core of person‐centred care is a close partnership with the patient, acknowledgement of the patient's narrative, and shared decision‐making [39]. This partnership with patients stretches from direct care to the design and development of healthcare systems. Aligning with this philosophy and incorporating principles of service design [24], engagement with stakeholders of various backgrounds was a key component of this iterative design process. The use of prototypes was central, as it facilitated and enabled stakeholder engagement throughout the process of developing more acceptable pictures. In the current study, the prototypes served as natural communication support in conversations with persons with aphasia. However, the use of prototypes also facilitated concrete and tangible feedback from healthcare professionals.

Lastly, service design has been successfully used to engage participants of older age and cognitive impairments [25]. Positioned along the continuum of stakeholder engagement in research, this study arguably aligns with a level between consultation and active participation [29]. Stakeholder engagement was embedded throughout the decision‐making process by involving a patient‐partner in consensus workshops. A higher degree of participation could have been achieved through joint co‐design workshops involving more stakeholders and the illustrator. Such collaborative engagement might have fostered a stronger sense of shared power among participants. Even so, it has been stated that engagement at every stage may not be realistic or even appropriate when collaborating with people of old age or with cognitive challenges [40].

This study adds to the limited research showing that people with aphasia can participate in collaborative design studies. In line with previous research [35, 36], the use of supportive communication strategies in the interviews was key to enable participation. We strongly advocate for persons with aphasia as collaborators in research in the future, since excluding this group from participating in co‐designing healthcare would essentially be to overlook close to a third of all stroke patients [2].

### 4.1 Limitations of Study

In this study, participants were purposively selected to contribute input and ideas on improvements. There was heterogeneity in age and gender; however, all persons with aphasia had functional reading ability and had relatively fluent speech. Even though they often spoke about the perspective of individuals with more severe aphasia, the first hand perspective of persons with severe aphasia was not included here. In future studies, a representation of greater variation of cultural backgrounds, as well as inclusion of family members and informal caretakers, may also increase the transferability of findings.

### 4.2 Clinical Implications

The presented findings may serve as principles for the future development of pictures to support health information. The addition of pictorial support to the PSC and the pre‐visit tool Stroke Health has the potential to support many patients in follow‐up after stroke. Even though the use of pictures is most common in communication with persons with aphasia, the use of pictures may also support understanding for patients with fatigue or cognitive impairment after stroke. Hence, a shift towards using patient‐directed questionnaires and information with pictures may benefit a broader group of individuals.

### 4.3 Future Directions

As this study is solely focused on the design process, future research needs to evaluate the usability, implementation and potential effects of the resource in clinical settings. Questions regarding whether, and to what extent, the pictures support patients in understanding the patient‐directed questions in Stroke Health and whether the pictures support communication between the patient and clinician during follow‐up with the PSC need to be investigated. Following an iterative design process, changes may be made when the pictures are tested in a clinical setting.

## 5 Conclusion

The results highlight several possible pitfalls and key elements to consider when developing pictorial support. This emphasises the need to continuously include stakeholders in the development of sustainable and person‐centred pictorial supports. The study contributes to the limited research demonstrating that persons with aphasia can participate in co‐design studies. The use of prototypes facilitated stakeholder engagement in both persons with aphasia and healthcare professionals. It also indicates that communication support goes beyond the use of pictures in conversation.

## Author Contributions

**Malin Bauer:** conceptualisation, formal analysis, investigation, methodology, writing – original draft. **Monica Blom Johansson:** conceptualisation, formal analysis, methodology, supervision, writing – review and editing, project administration. **Ellika Schalling:** conceptualisation, formal analysis, supervision, writing – review and editing, methodology. **Emma Kjörk:** conceptualisation, formal analysis, investigation, methodology, supervision, writing – review and editing, project administration.

## Ethics Statement

Ethical approval was obtained by the Swedish Ethical Review 2017‐08‐29; 556‐17 and 2022‐01‐11; 2021‐06723‐02.

## Conflicts of Interest

The authors declare no conflicts of interest.

## Supporting information

Appendix1_HE_accepted‐251015.

Appendix2_HE_accepted‐251015.

## Data Availability

Due to the nature of the research, supporting data are not available for ethical reasons. The participants of this study did not give written consent for their data to be shared publicly.
