# Supplementary material for: Identification and Applications of Key Elements in the Development of Pictorial Support for People With Aphasia After Stroke: A Co‐Design Approach
Source: Health Expect. 2025 Oct 29;28(6):e70478. doi: 10.1111/hex.70478 (PMC12570041; doi:10.1111/hex.70478)
Supplement: Supplementary file 1 — Appendix1_HE_accepted‐251015. [file HEX-28-e70478-s001.pdf]

Follow-up after stroke and TIA
- the Post-stroke checklist

Picture supported version

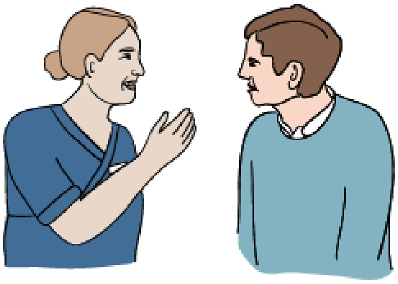

This picture-assisted version of the Post-stroke checklist is a communication aid for follow-up after stroke and TIA.

The pictures are a supplement to the questions to clarify what the question is about. You may also need to adapt the wording of the questions to the person in front of you. In case of repeated follow-up: change " since your stroke" to "last assessment".

People with language and cognitive difficulties after a brain injury are often in need of communication support in conversations, both to support understanding and expressiveness.

Using communication support for patients with communication difficulties is therefore an important step in ensuring safe and secure care.

The Post-stroke checklist is endorsed by the World Stroke Organization and adapted from Philp I, et al. 2013. This picture supported version has been developed in collaboration with the national work group for stroke (NAG) and adapted within research at Göteborgs, and Uppsala universitet. Bauer M et al., (2025) Heath Expectations.
https://vardpersonal.1177.se/om-1177-for-vardpersonal/about-us/

Pre-visit forms for patients are avaliable at: https://www.gu.se/neurovetenskap-fysiologi/strokehalsatm-uppfoljning-efter-stroke

Illustration: Annie Simlund, Västra Götalandsregionen. The illustrations may be shared, copied and redistributed but not used for commercial purposes. The originator must be stated in a reasonable manner. Bauer M et al., (2025) Health Expectations. Version 1.0.

Register

	**Post-stroke checklist**
1	Secondary prevention
2	Aktivity of daily living (ADL)
3	Mobility
4	Spasticity
5	Pain
6	Incontinence
7	Communication
8	Mood
9	Cognition
10	Life after stroke
11	Relationsship with family
	Additional questions:
12	Fatigue
13	Oral health, swallowing and nutrition
14	Other challenges
	**Complementary questions**
1a-e	Lifestyle habits
2 & 3	Samples, blood pressure and EKG
4	Drugs
5	Driving
6	Fire arms
7	Sickleave
8	Follow-up appointment

Illustration: Annie Simlund, Västra Götalandsregionen. The illustrations may be shared, copied and redistributed but not used for commercial purposes. The originator must be stated in a reasonable manner. Bauer M et al., (2025) Health Expectations. Version 1.0.

1

Secondary prevention

Since your stroke, have you received any advice on health related lifestyle changes or medications for preventing another stroke?

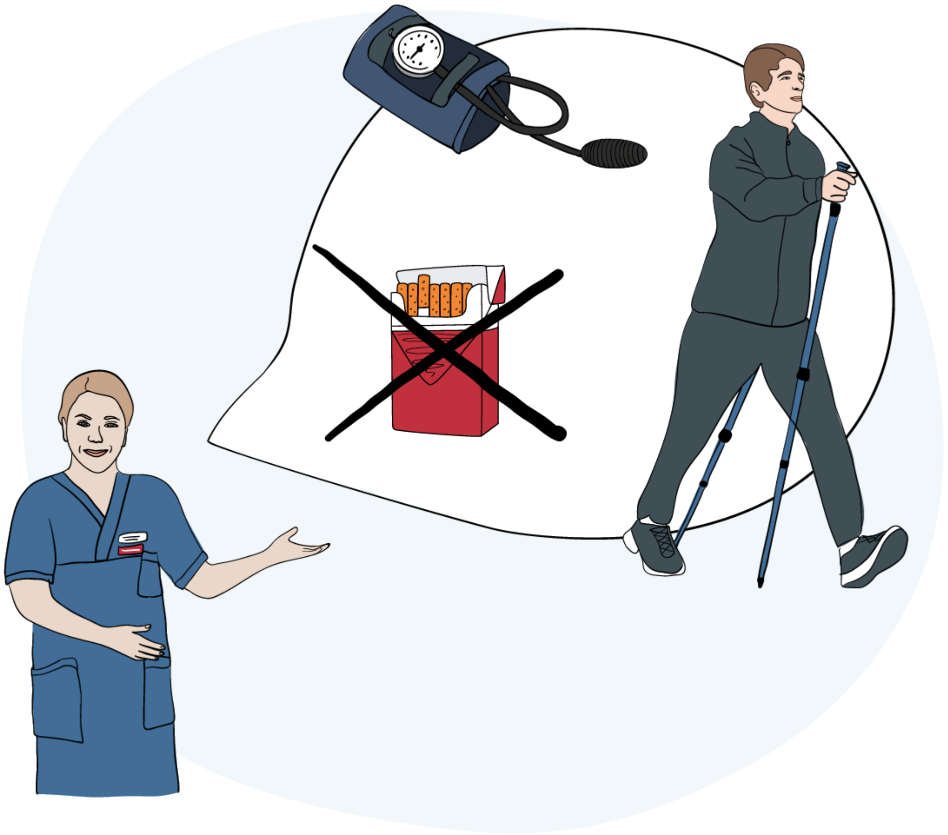

Yes

No

Illustration: Annie Simlund, Västra Götalandsregionen. The illustrations may be shared, copied and redistributed but not used for commercial purposes. The originator must be stated in a reasonable manner. Bauer M et al., (2025) Health Expectations. Version 1.0.

2 Activities of daily living (ADL)

Since your stroke, are you finding it **more** difficult to take care of yourself?

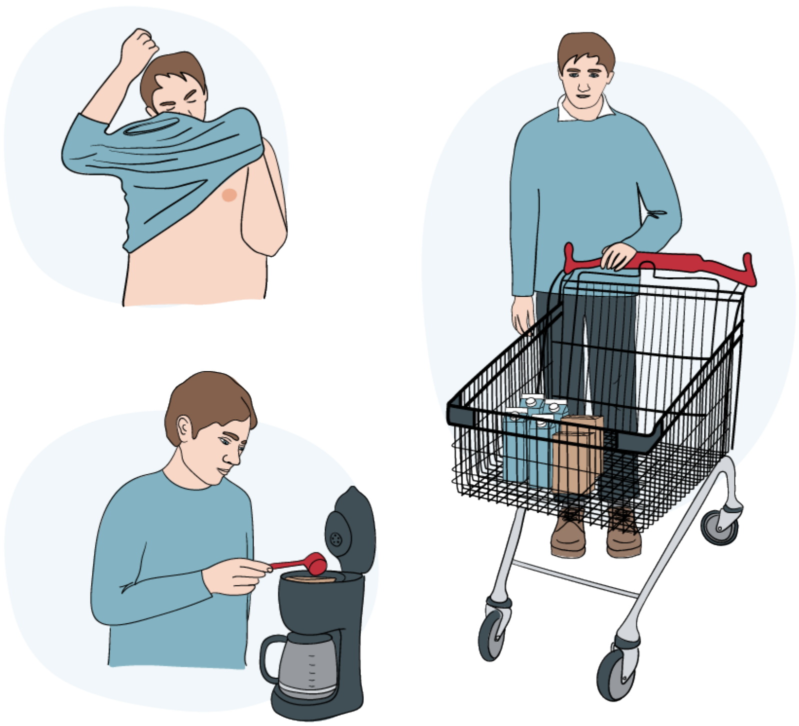

Yes

No

Illustration: Annie Simlund, Västra Götalandsregionen. The illustrations may be shared, copied and redistributed but not used for commercial purposes. The originator must be stated in a reasonable manner. Bauer M et al., (2025) Health Expectations. Version 1.0.

3

Mobility

Since your stroke, are you finding it **more** difficult to walk or move safely from bed to chair?

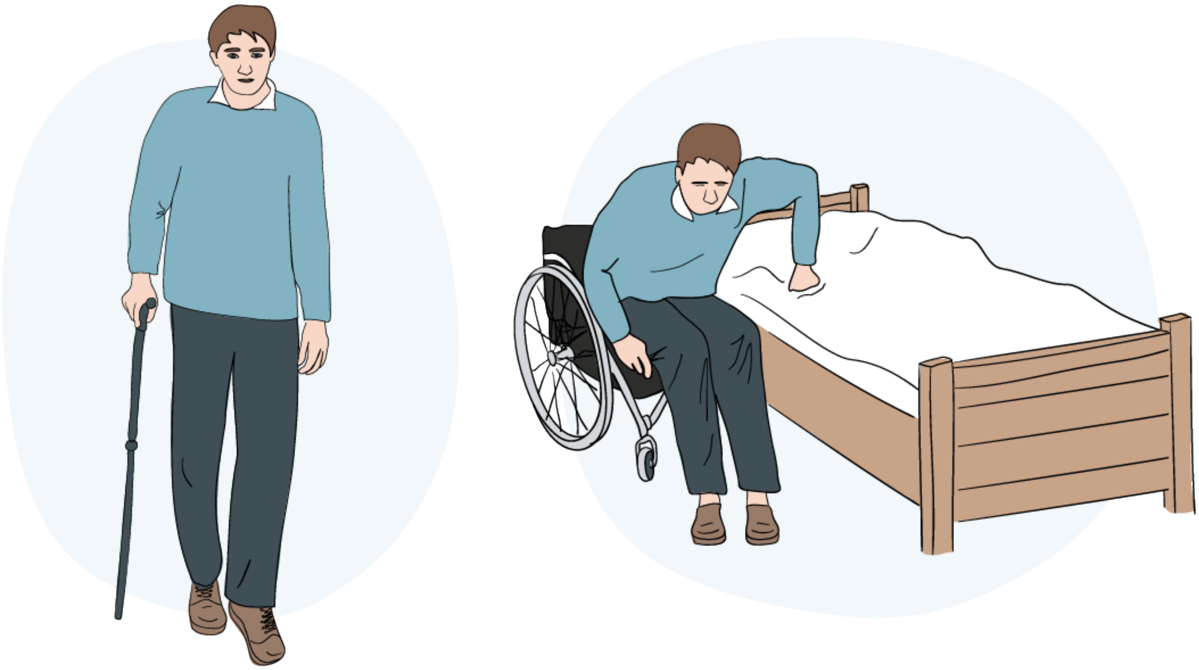

Yes

No

Illustration: Annie Simlund, Västra Götalandsregionen. The illustrations may be shared, copied and redistributed but not used for commercial purposes. The originator must be stated in a reasonable manner. Bauer M et al., (2025) Health Expectations. Version 1.0.

4

Spasticity

Since your stroke, do you have **increasing** stiffness in your arms, hands, and/or legs?

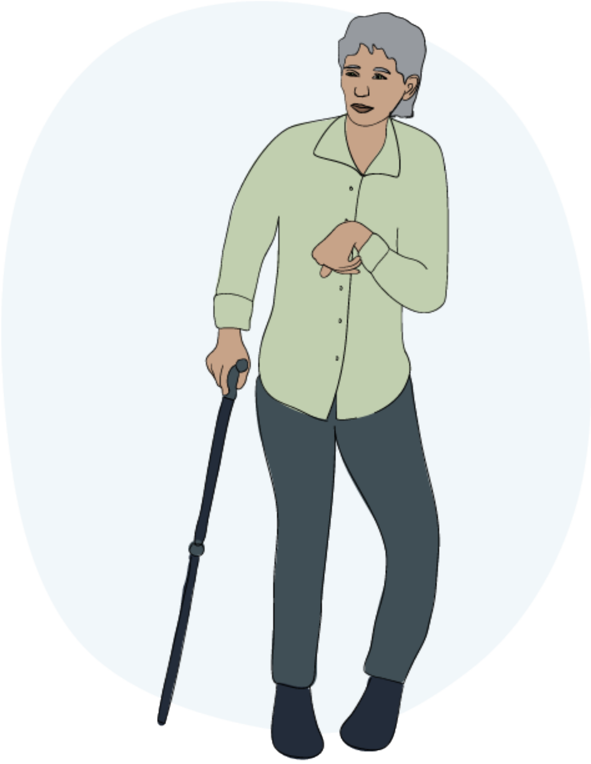

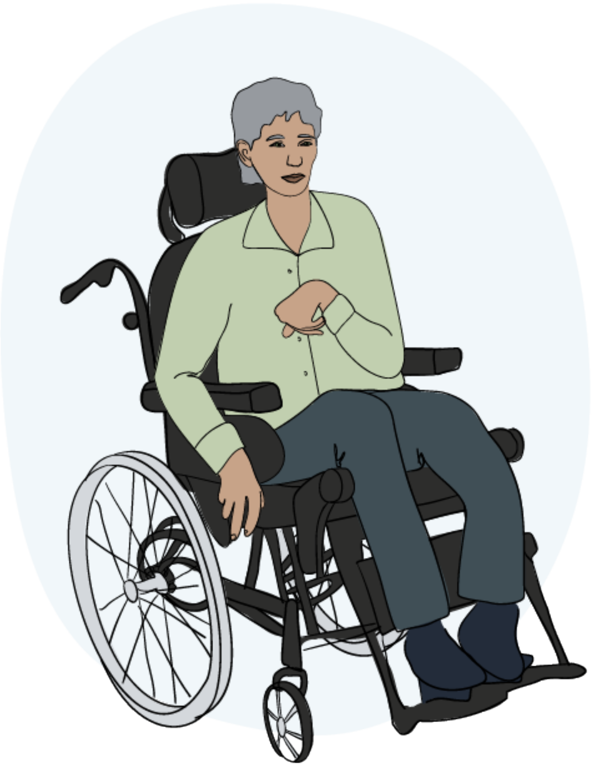

Yes

No

Illustration: Annie Simlund, Västra Götalandsregionen. The illustrations may be shared, copied and redistributed but not used for commercial purposes. The originator must be stated in a reasonable manner. Bauer M et al., (2025) Health Expectations. Version 1.0.

5

Pain

Since your stroke, do you have any **new** pain?

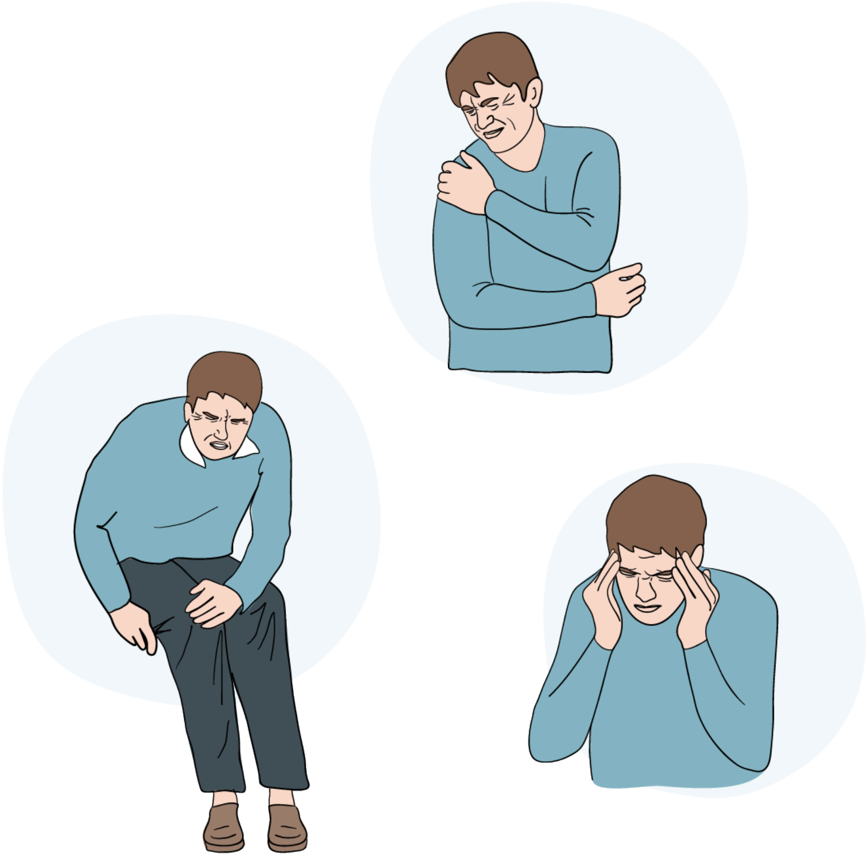

Yes

No

Illustration: Annie Simlund, Västra Götalandsregionen. The illustrations may be shared, copied and redistributed but not used for commercial purposes. The originator must be stated in a reasonable manner. Bauer M et al., (2025) Health Expectations. Version 1.0.

6

Incontinence

Since your stroke, are you having **<u>more</u>** of a problem controlling your bladder or bowels?

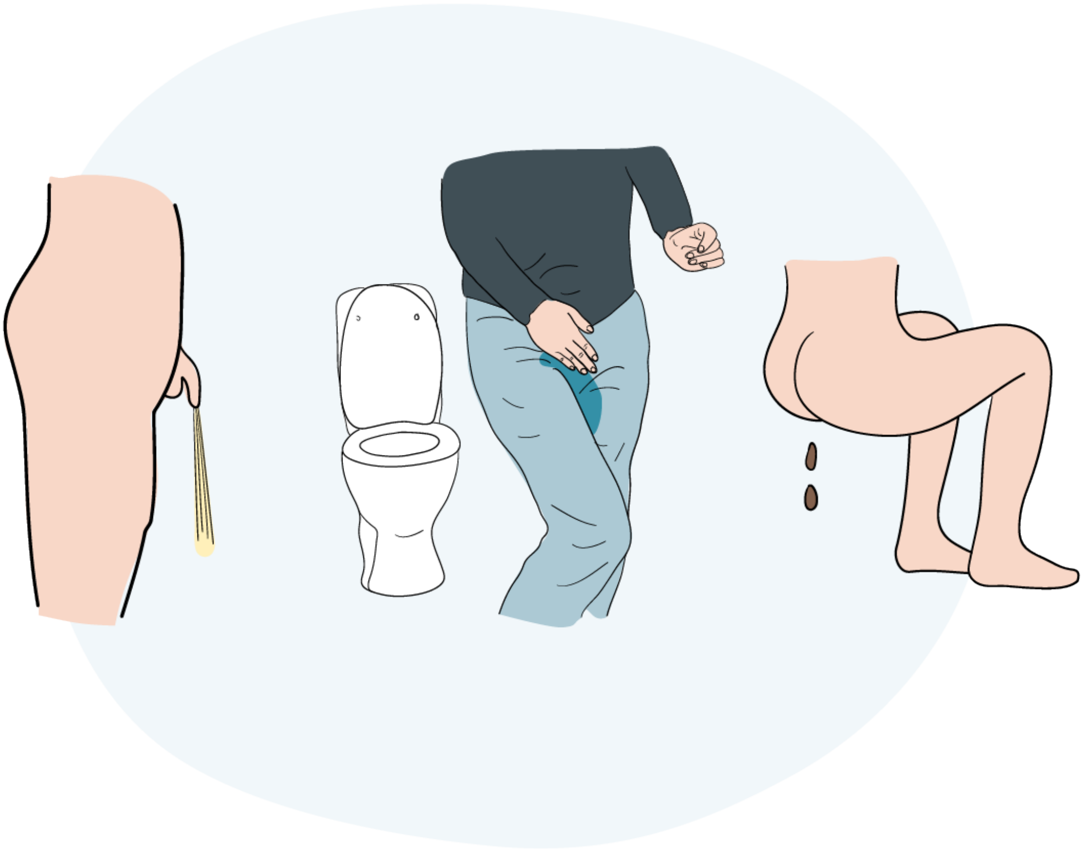

Yes

No

Illustration: Annie Simlund, Västra Götalandsregionen. The illustrations may be shared, copied and redistributed but not used for commercial purposes. The originator must be stated in a reasonable manner. Bauer M et al., (2025) Health Expectations. Version 1.0.

7 Communication

Since your stroke, are you finding it **<u>more</u>** difficult to communicate with others?

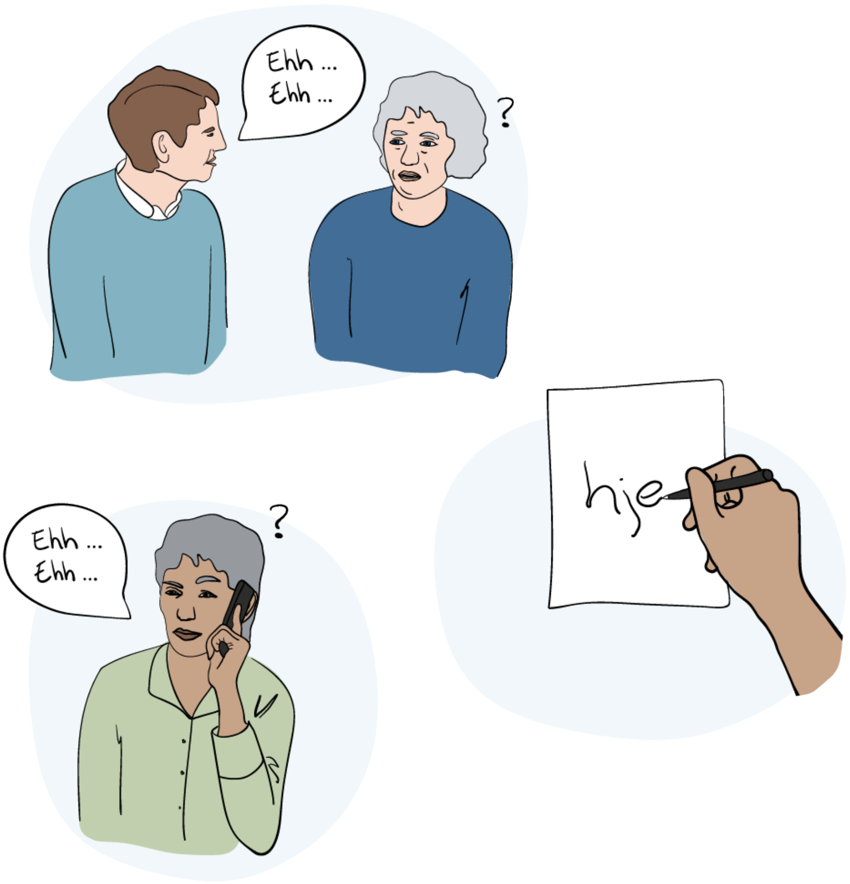

Yes

No

Illustration: Annie Simlund, Västra Götalandsregionen. The illustrations may be shared, copied and redistributed but not used for commercial purposes. The originator must be stated in a reasonable manner. Bauer M et al., (2025) Health Expectations. Version 1.0.

8

Mood

Since your stroke, do you feel **more** anxious or depressed?

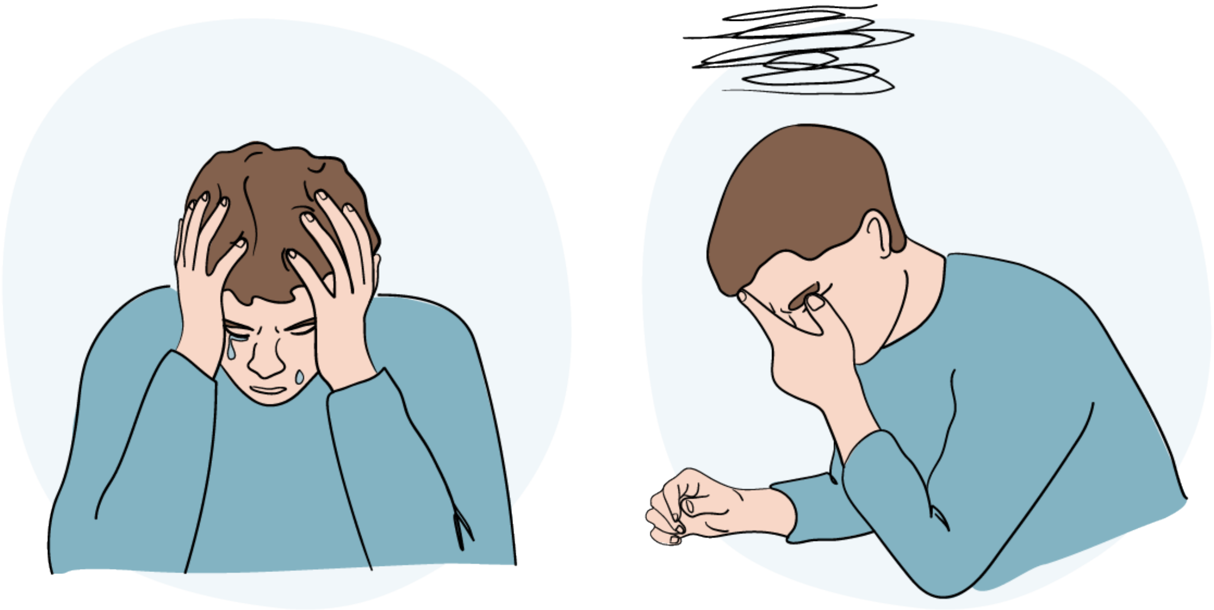

Yes

No

Illustration: Annie Simlund, Västra Götalandsregionen. The illustrations may be shared, copied and redistributed but not used for commercial purposes. The originator must be stated in a reasonable manner. Bauer M et al., (2025) Health Expectations. Version 1.0.

9 Cognition

Since your stroke, are you finding it **more** difficult to think, concentrate, or remember things?

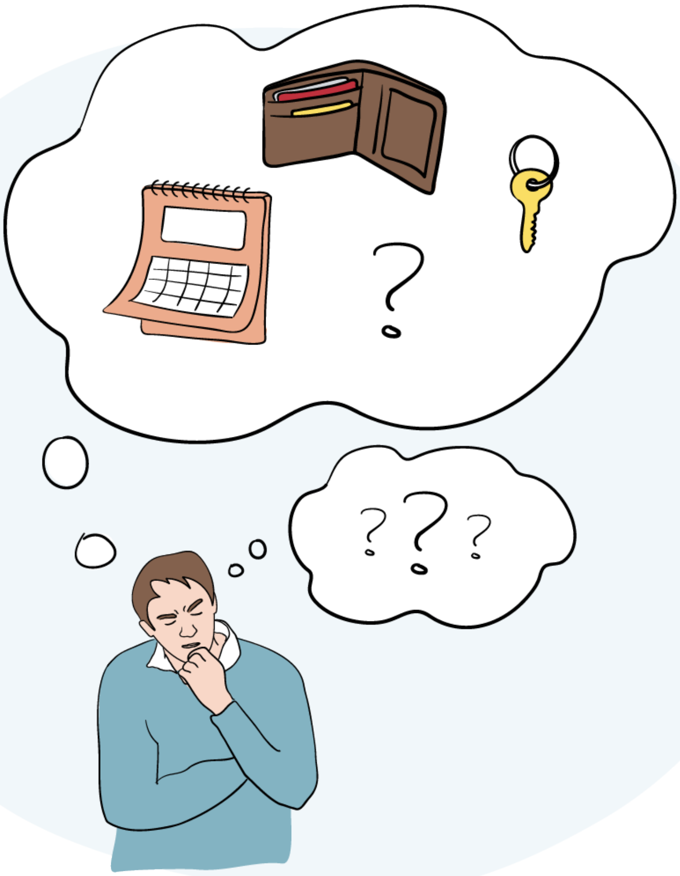

Yes

No

Illustration: Annie Simlund, Västra Götalandsregionen. The illustrations may be shared, copied and redistributed but not used for commercial purposes. The originator must be stated in a reasonable manner. Bauer M et al., (2025) Health Expectations. Version 1.0.

10 Life after stroke

Since your stroke, are you finding things important to you **more** difficult to carry out (e.g. leisure activities, hobbies, work, as well as relationships with loved ones, where appropriate)?

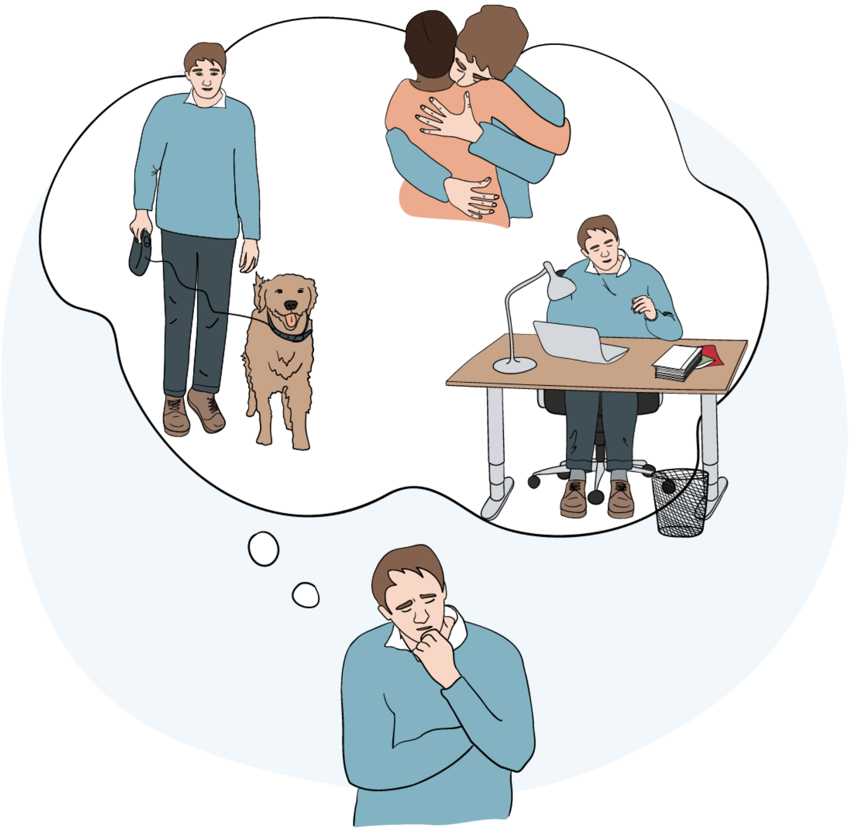

Yes

No

Illustration: Annie Simlund, Västra Götalandsregionen. The illustrations may be shared, copied and redistributed but not used for commercial purposes. The originator must be stated in a reasonable manner. Bauer M et al., (2025) Health Expectations. Version 1.0.

11 **Relationship with family**

Since your stroke, has your relationship with your family become **more** difficult or stressed?

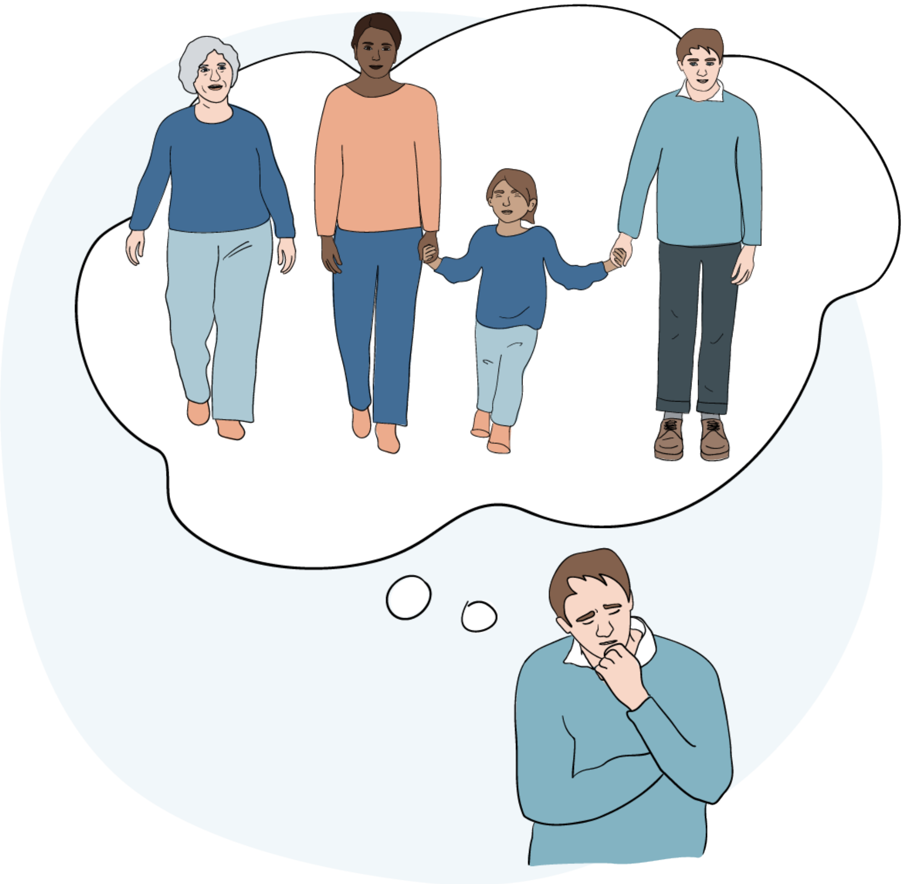

Yes

No

Illustration: Annie Simlund, Västra Götalandsregionen. The illustrations may be shared, copied and redistributed but not used for commercial purposes. The originator must be stated in a reasonable manner. Bauer M et al., (2025) Health Expectations. Version 1.0.

12

Fatigue

Since your stroke, do you experience **increased** fatigue that affects your ability to exercise or perform other activities?

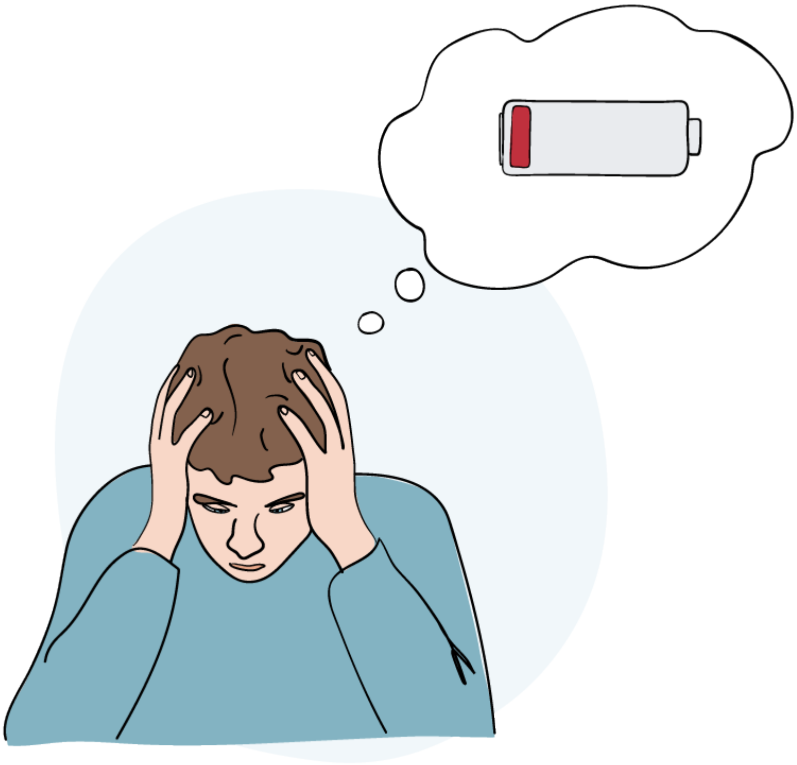

Yes

No

Illustration: Annie Simlund, Västra Götalandsregionen. The illustrations may be shared, copied and redistributed but not used for commercial purposes. The originator must be stated in a reasonable manner. Bauer M et al., (2025) Health Expectations. Version 1.0.

13

Oral health, swallowing and nutrition

Since your stroke, do you find it **more** difficult to manage your oral hygiene, to eat or swallow?

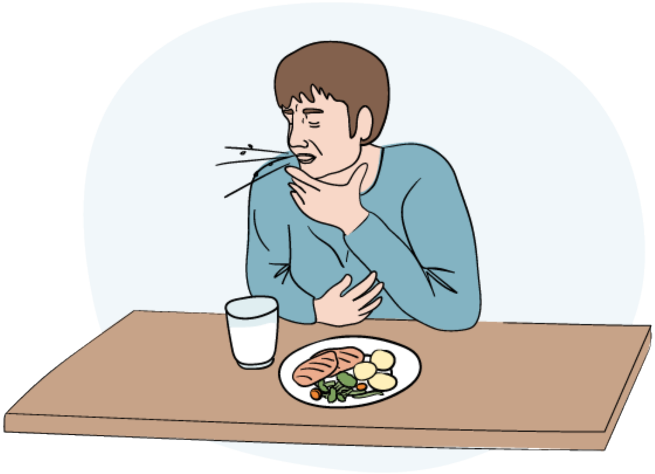

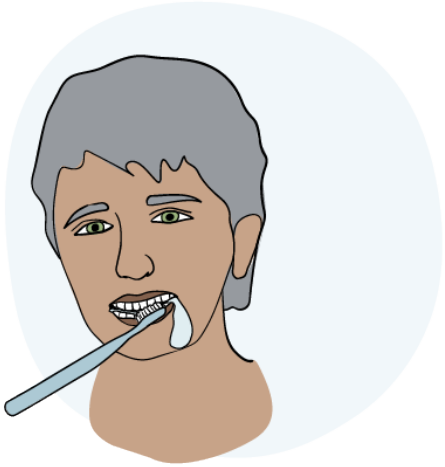

Yes

No

Illustration: Annie Simlund, Västra Götalandsregionen. The illustrations may be shared, copied and redistributed but not used for commercial purposes. The originator must be stated in a reasonable manner. Bauer M et al., (2025) Health Expectations. Version 1.0.

14 Other challenges

Do you have any other concerns after your stroke that affect your recovery or cause you difficulties?

(e.g., balanse, visual, attention, sexual life, fears)

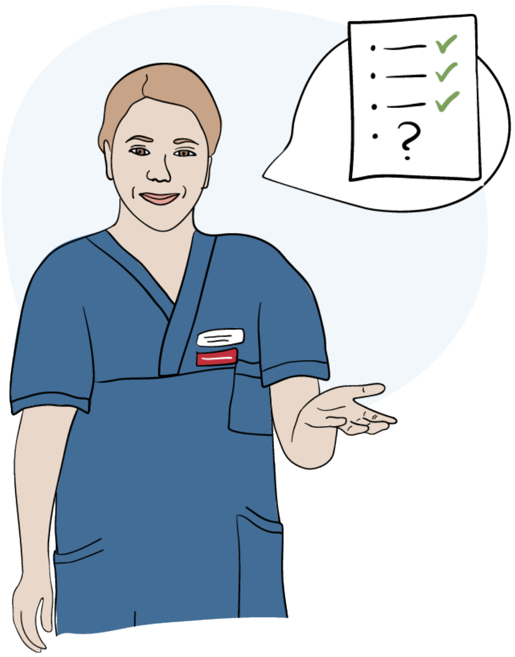

Yes

No

Illustration: Annie Simlund, Västra Götalandsregionen. The illustrations may be shared, copied and redistributed but not used for commercial purposes. The originator must be stated in a reasonable manner. Bauer M et al., (2025) Health Expectations. Version 1.0.

Complementary questions to the Post-stroke checklist

To be answered at a team round or a doctors appoinment.

1a

Lifestyle habits

Do you smoke?

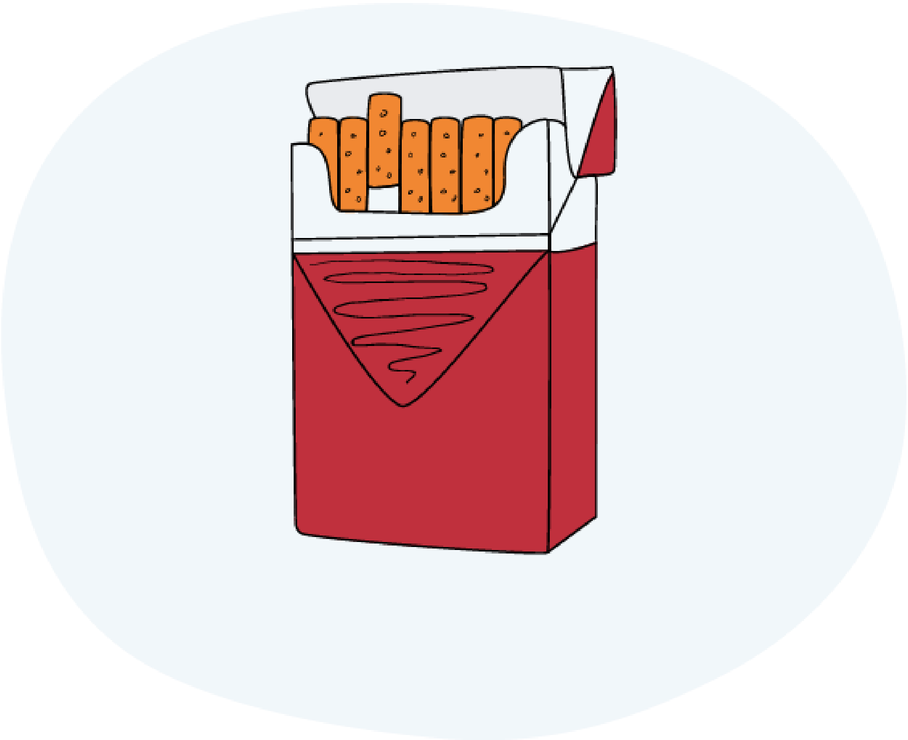

Yes

No

Illustration: Annie Simlund, Västra Götalandsregionen. The illustrations may be shared, copied and redistributed but not used for commercial purposes. The originator must be stated in a reasonable manner. Bauer M et al., (2025) Health Expectations. Version 1.0.

1b

Lifestyle habits

Do you drink alcohol?

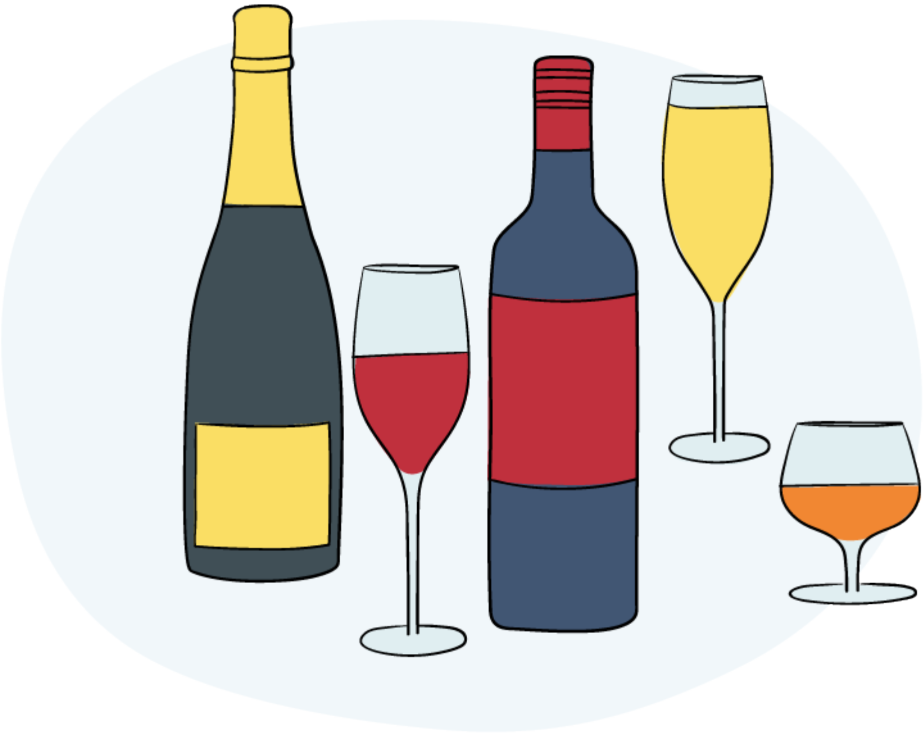

Yes

No

Illustration: Annie Simlund, Västra Götalandsregionen. The illustrations may be shared, copied and redistributed but not used for commercial purposes. The originator must be stated in a reasonable manner. Bauer M et al., (2025) Health Expectations. Version 1.0.

1c

Lifestyle habits

Are you physically active?

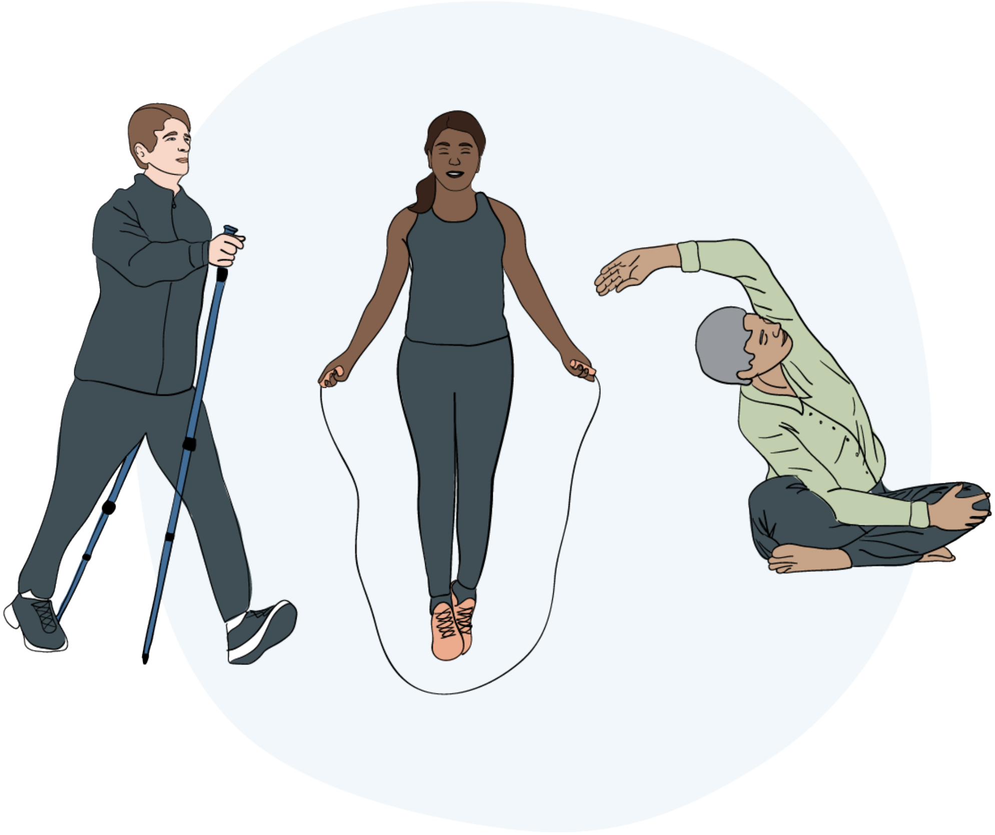

Yes

No

Illustration: Annie Simlund, Västra Götalandsregionen. The illustrations may be shared, copied and redistributed but not used for commercial purposes. The originator must be stated in a reasonable manner. Bauer M et al., (2025) Health Expectations. Version 1.0.

1d

Lifestyle habits

Do you have good eating habits?

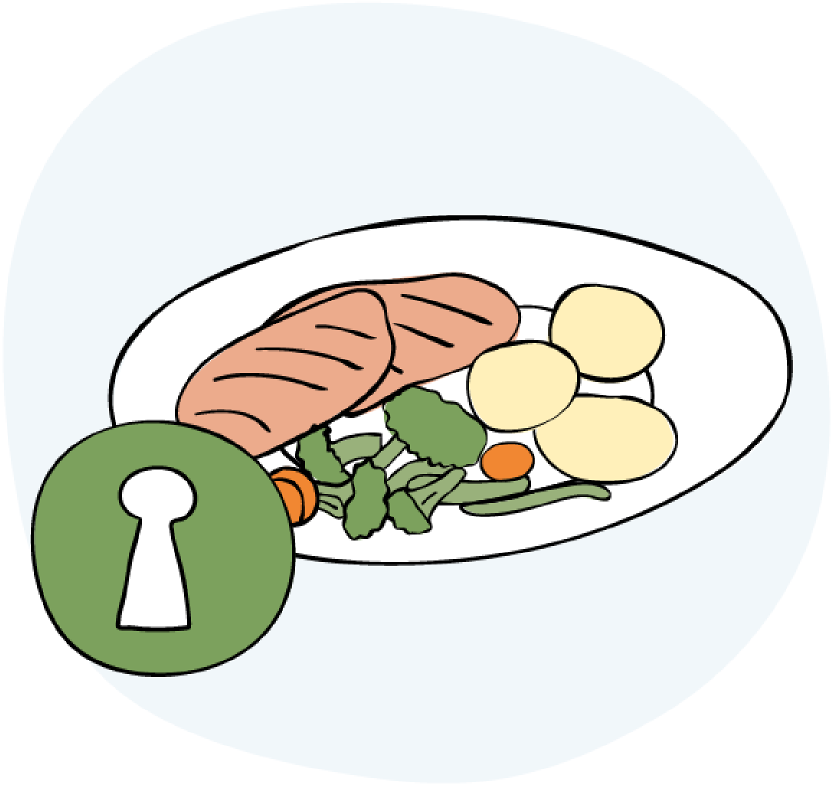

Yes

No

Illustration: Annie Simlund, Västra Götalandsregionen. The illustrations may be shared, copied and redistributed but not used for commercial purposes. The originator must be stated in a reasonable manner. Bauer M et al., (2025) Health Expectations. Version 1.0.

1e

Lifestyle habits

Do you experience stress?

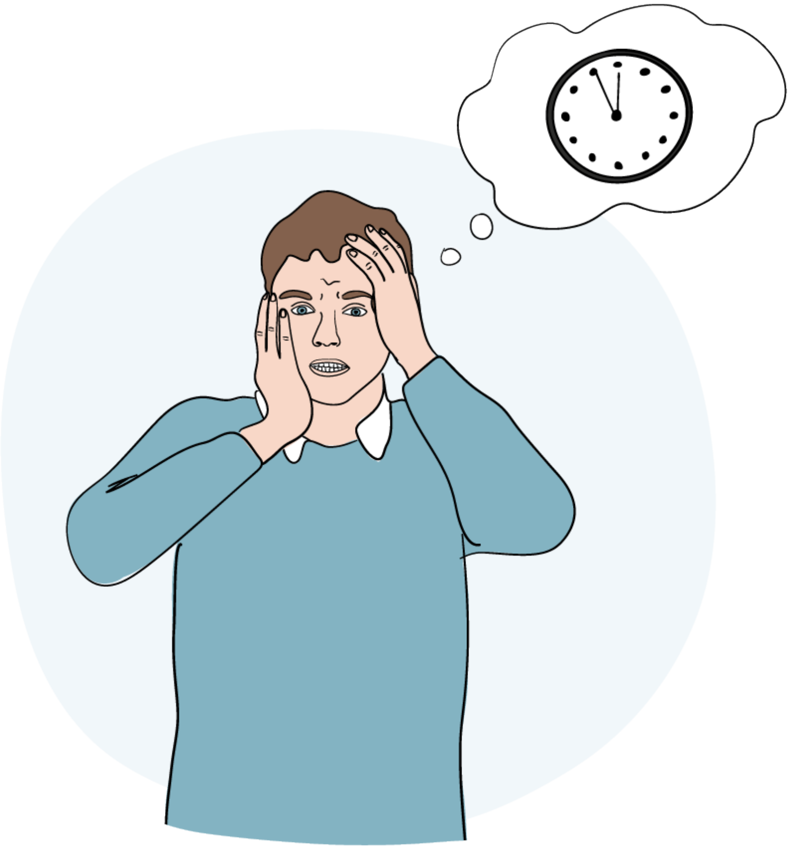

Yes

No

Illustration: Annie Simlund, Västra Götalandsregionen. The illustrations may be shared, copied and redistributed but not used for commercial purposes. The originator must be stated in a reasonable manner. Bauer M et al., (2025) Health Expectations. Version 1.0.

2 3

Samples

Sampling, blood pressure and EKG

(Lab samples: blood status, electrocytes, creatine, blood fats, ALAT, lipids, B-glucose)

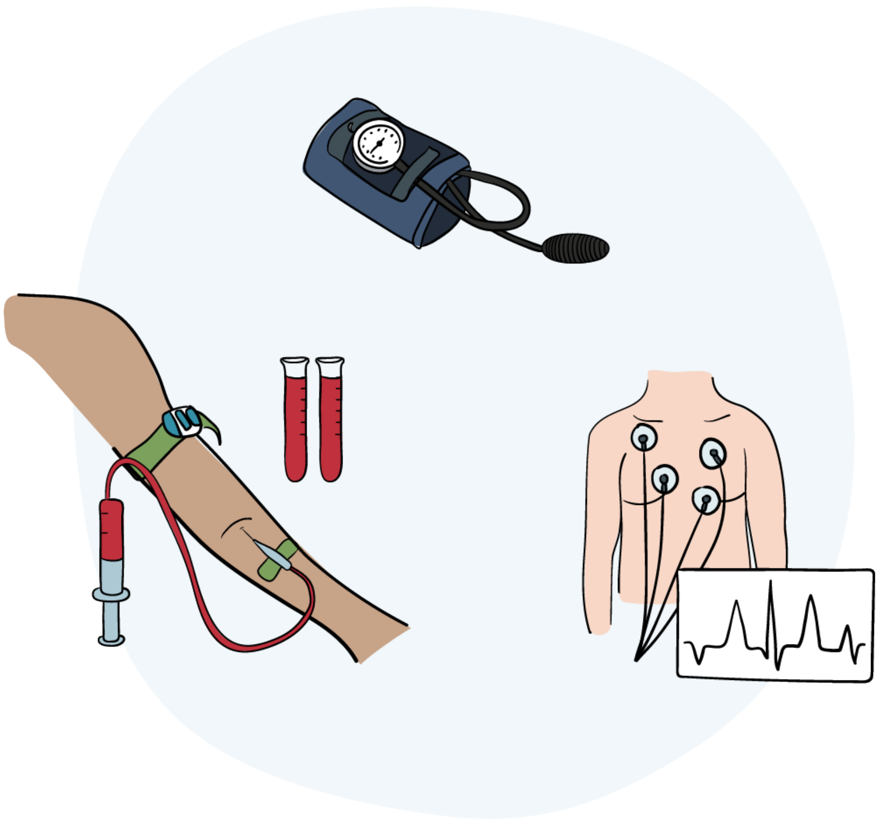

Yes

No

Illustration: Annie Simlund, Västra Götalandsregionen. The illustrations may be shared, copied and redistributed but not used for commercial purposes. The originator must be stated in a reasonable manner. Bauer M et al., (2025) Health Expectations. Version 1.0.

4

Drugs

Do you have side effects of from your medications?

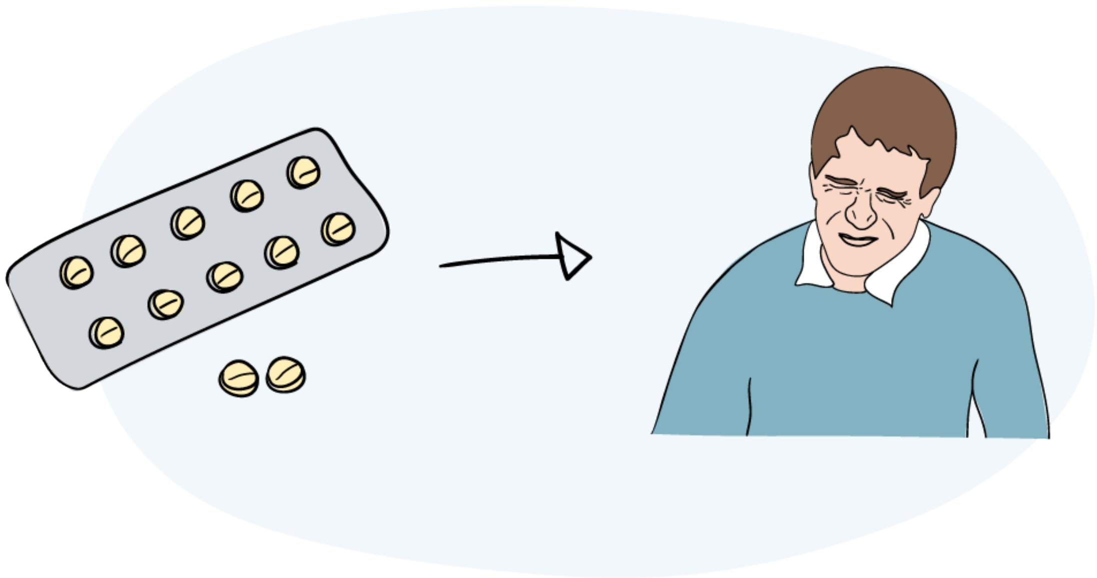

Yes

No

Illustration: Annie Simlund, Västra Götalandsregionen. The illustrations may be shared, copied and redistributed but not used for commercial purposes. The originator must be stated in a reasonable manner. Bauer M et al., (2025) Health Expectations. Version 1.0.

5 Driving

Do you have a driving license?

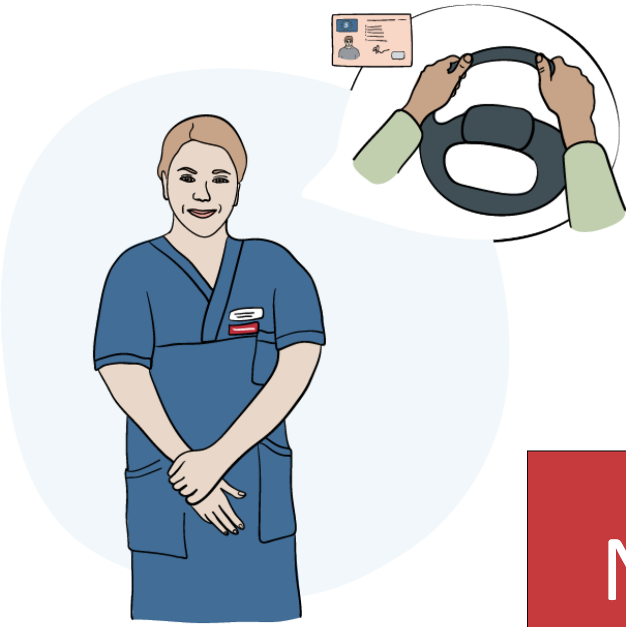

Yes

No

Do you drive?

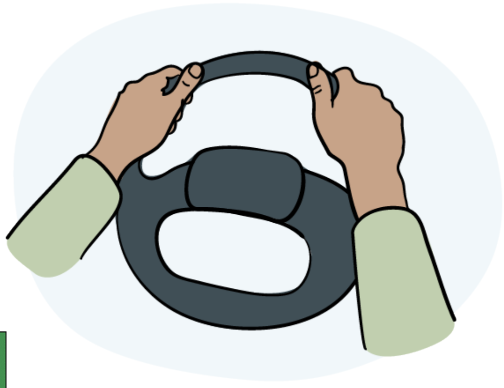

Yes

No

Illustration: Annie Simlund, Västra Götalandsregionen. The illustrations may be shared, copied and redistributed but not used for commercial purposes. The originator must be stated in a reasonable manner. Bauer M et al., (2025) Health Expectations. Version 1.0.

6

Firearms

Do you have a gun license?

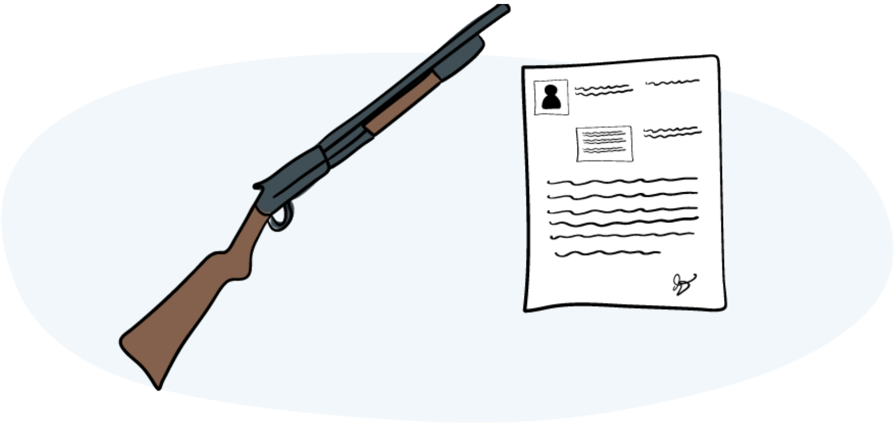

Yes

No

Do you own firearms?

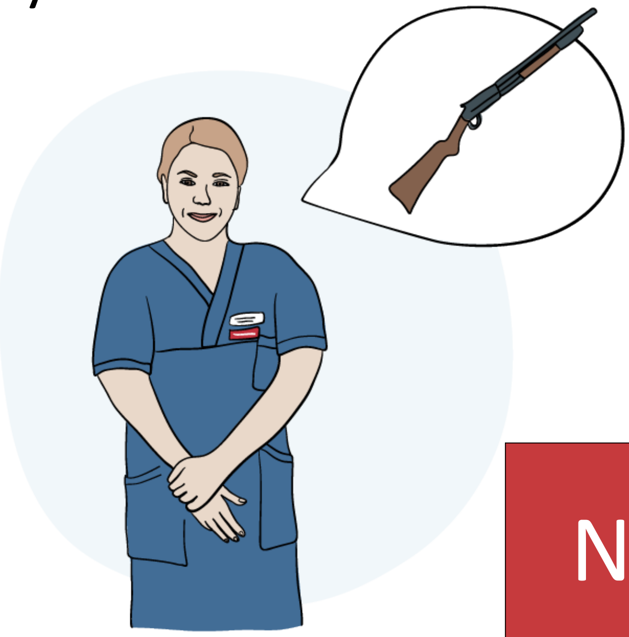

Yes

No

Illustration: Annie Simlund, Västra Götalandsregionen. The illustrations may be shared, copied and redistributed but not used for commercial purposes. The originator must be stated in a reasonable manner. Bauer M et al., (2025) Health Expectations. Version 1.0.

7

Sick-leave

Do you work professionally?
Are you on sick-leave?

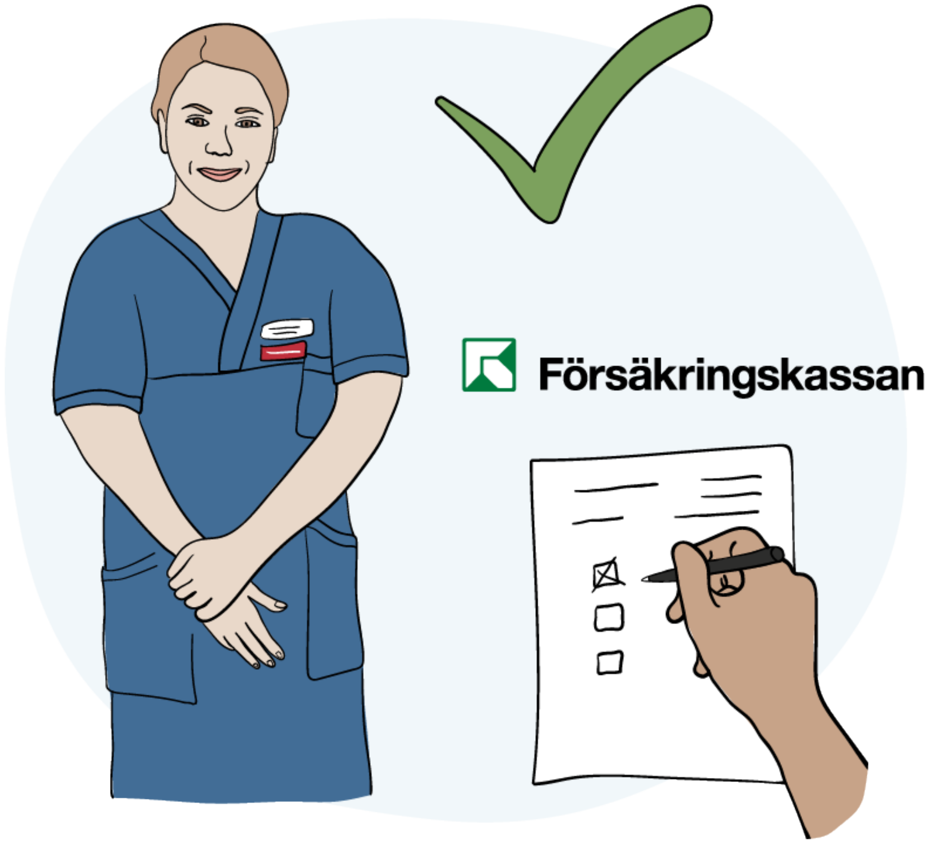

Yes

No

Illustration: Annie Simlund, Västra Götalandsregionen. The illustrations may be shared, copied and redistributed but not used for commercial purposes. The originator must be stated in a reasonable manner. Bauer M et al., (2025) Health Expectations. Version 1.0.

8

Follow-up

Has a new time for follow up been booked?

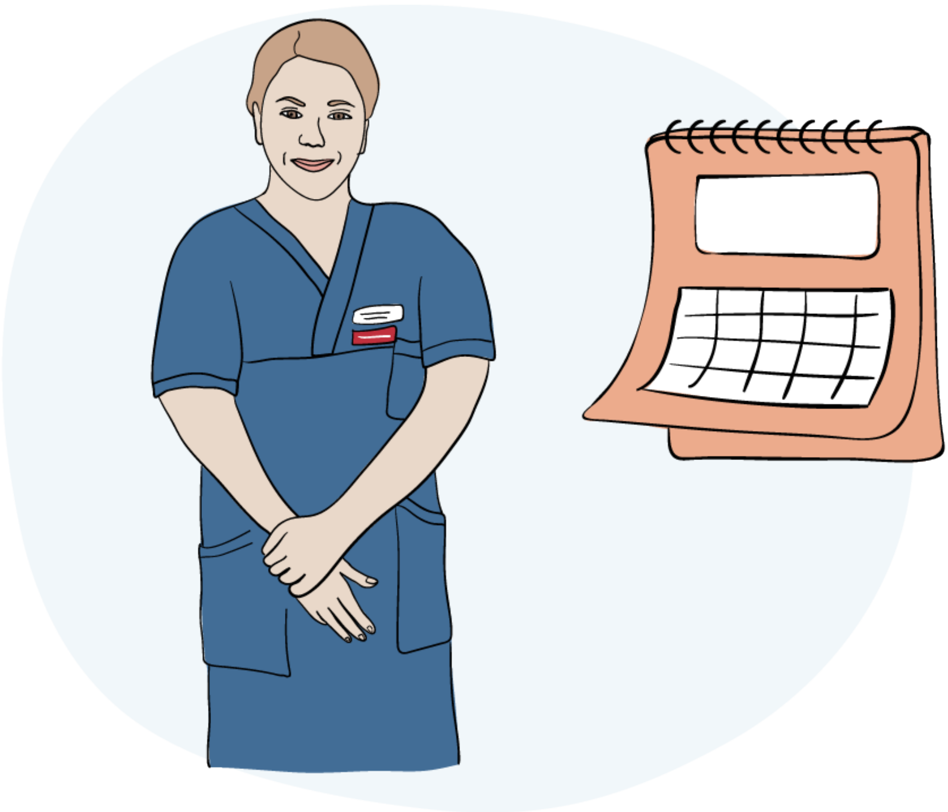

Yes

No

Illustration: Annie Simlund, Västra Götalandsregionen. The illustrations may be shared, copied and redistributed but not used for commercial purposes. The originator must be stated in a reasonable manner. Bauer M et al., (2025) Health Expectations. Version 1.0.
